# At Washington

**Published:** 1887-10

**Authors:** 


### AT WASHINGTON.

The members of Section XVII from abroad, as a body, reflected honor upon the profession of the countries of which they were representative. Prof. Dr. Busch, of the University of Berlin, was detained upon the ocean by stormy weather, and did not arrive until the third day. But he made a very favorable impression, and was most enthusiastically received when he presented the very remarkable collection of pathological anomalies in elephants' teeth before the Section.

Dr. J. E. Grevers, of Amsterdam, one of the State Examiners for Holland; Dr. Ernst Sjoberg, of Stockholm; Dr. W. B. McLeod, of Edinburgh; Dr. R. T. Stack, of Dublin; Drs. Woodhouse and Mummery, of England, and many others whose names amid the confused memories of the week have now escaped, but whose cheering presence was perceptibly felt at the meeting, were thrice welcome as giving a truly international character to the sessions, and as affording an opportunity for a return of the hospitalities which were so lavishly displayed for the benefit of the very much larger American delegation which attended the meeting in London.

